# Strategic Wholesale Pricing for an Incumbent Supplier Facing with a Competitive Counterpart

**DOI:** 10.1155/2014/604634

**Published:** 2014-10-14

**Authors:** Jianwu Sun

**Affiliations:** Department of Mathematics and Information Science, Binzhou University, Binzhou, Shandong 256603, China

## Abstract

We introduce a wholesale pricing strategy for an incumbent supplier facing with a competitive counterpart. We propose a profit function which considers both the present loss and future loss from a wholesale price and then study the optimal wholesale prices for different objectives about this profit function for the incumbent supplier. First, we achieve an optimal wholesale price for the incumbent supplier to maximize his expected profit. Then, to reduce the risk originating from the fluctuation in the competitive supplier's wholesale price, we integrate the conditional value-at-risk (CVaR) measure in financial risk management into this study and derive an optimal wholesale price to maximize CVaR about profit for the incumbent supplier. Besides, the properties of the two optimal wholesale prices are discussed. Finally, some management insights are suggested for the incumbent supplier in a competitive setting.

## 1. Introduction

In recent years, the study about the supplier's wholesale pricing strategies develops an interesting topic and has attracted the attentions of many researchers. In some literature, it is often supposed that the supplier is in the predominant position of deciding the wholesale price; that is, the supplier is a leader to announce a wholesale price and the retailer acts as a follower to give an order quantity; see Bernstein and Federgruen [1], Choudhary et al. [2], and Ma et al. [3]. However, an incumbent supplier in reality often confronts the competitions from other potential entrant suppliers, and then the incumbent supplier needs to give a “competitive wholesale price” to continue the cooperation with his retailers. Moreover, although a retailer needs to stick with an incumbent supplier for a specified period of time, he often switches to other competitive suppliers providing lower wholesale prices and/or better customer service when the time is due. Then, facing a highly competitive environment, some incumbent suppliers are more concerned to build retailer loyalty and continue the cooperation with the retailers by providing products of high quality and better service for his customers. Sometimes, however, it is difficult for an incumbent supplier to win his customers in the fierce competition only by the advantage of product quality and service level, and price advantage is an important factor to win such a competition, especially for the competing suppliers that provide the same/similar products. Then, to build retailer loyalty and gain more profits from the sustained cooperation with a retailer, adopting an effective pricing strategy is very important for an incumbent supplier in a competitive setting. Here, the incumbent supplier's pricing strategy cannot be an aimless price fight but will be a rational reaction to both his competitor actions and the retailer's preference.

This paper thus considers a wholesale pricing strategy for an incumbent supplier who confronts with the competition from a competitive supplier. It is supposed that there exists an order contract between the incumbent supplier and the retailer, but the retailer can switch to the competitive supplier when this contract is due if the competitive supplier provides a lower wholesale price. Then, if the incumbent supplier wants to increase his profit in the current deal and extend the order contract with the retailer at the same time, he must give a proper wholesale price to balance the two objectives. On the one hand, if the incumbent supplier gives a low wholesale price to encourage the retailer to extend the contract, he may lose some profit in the current deal. On the other hand, if the incumbent supplier gives a high wholesale price to improve his profit in the current deal, the retailer may switch to the competitive supplier when the contract is due and the incumbent supplier thus loses the retailer. Then, the incumbent supplier must give balance between a low wholesale price and a high wholesale price. In view of this critical issue, this paper introduces a profit function, which considers both the present loss and the future loss from a wholesale price of the incumbent supplier. Then, we discuss the optimal wholesale prices to optimize different objectives about this profit function for the incumbent supplier. First, we achieve an optimal wholesale price for the incumbent supplier who aims to maximize his expected profit. However, such an expected performance measure is still insufficient for some incumbent suppliers because it fails to control/reduce the risk originating from the fluctuation in the competitive supplier's wholesale price. Then, to reduce such a risk, we integrate the CVaR measure into this study and derive an optimal wholesale price to maximize the CVaR about profit for the incumbent supplier. This optimal wholesale price can maximize the expectation of the profit that is below a given target level for the incumbent supplier. Besides, the properties of the two optimal wholesale prices are discussed as well.

Our paper thus contributes to the growing researches on the wholesale pricing strategies of the suppliers in a competitive setting. Since the proposed profit function considers both the present loss and the future loss from a wholesale price of the incumbent supplier, the optimal wholesale price to maximize the expected profit can maximize the incumbent supplier's total profit obtained from the current and future cooperations with his retailer. Specifically, by adopting the CVaR measure, the optimal wholesale price to maximize the CVaR about profit can help the incumbent supplier to control/reduce the risk originating from the fluctuation in the competitive supplier's wholesale price. This also leads to insights for the risk management of the suppliers in a competitive setting.

The rest of this paper is organized as follows. In the following section, we review some related research work.  Section 3 presents the model and studies the optimal wholesale prices for different objectives for the incumbent supplier, with conclusions given in Section 4.

## 2. Literature Review

Due to space limitation, we review the most related studies about the wholesale pricing strategy of a supplier in a competitive environment.

Recently, with the development of globalization, competitions in supply chains have received more and more attention in operations management study. Thus, devising efficient pricing strategies for a supplier to compete with other suppliers comes into focus. For example, Dai et al. [4] studied the pricing strategies of multiple suppliers providing the same service in competition for a common pool of customers in a revenue management context. It is supposed that these suppliers all want to maximize their own profits by setting prices to attract potential retailers and game theory is applied to analyze this problem. Sohn et al. [5] derived a dynamic pricing model for the competitive supplier under a mobile phone set to maximize his profit. Both pricing changes of the competitive supplier and his competitors are discussed, and scenario analysis is also performed to find the optimal pricing policy for the competitive supplier. Xiao and Qi [6] developed an adverse selection model for the two-stage supply chain that consists of a retailer, an incumbent supplier, and a potential outside entrant supplier. They investigated how the incumbent supplier can strategically maximize his profit by devising a wholesale pricing policy when facing the potential entrant supplier. Li et al. [7] investigated the sourcing strategy of a retailer and the pricing strategies of two competitive suppliers in a supply chain. They derived the sufficient condition for the existence of an equilibrium price for the competitive suppliers and introduced a coordination mechanism to maximize the profits of the competitive suppliers. Xia [8] studied the competitive strategies between two coexisting suppliers in a two-echelon supply chain, and pricing strategies for different retailer groups are suggested to the competitive suppliers. Wang et al. [9] studied and compared the performances of two different markup pricing strategies in a supply chain with a dominant retailer and two competitive suppliers, and some interesting results are achieved.

In general, most of the existing wholesale pricing strategies focus on attaining a supplier's target in terms of profit improvement or cost reduction, while the importance of continuing the cooperation with a retailer is often neglected. Evidently, continuing the cooperation with a retailer can never be ignored in devising the wholesale pricing strategies for the incumbent supplier in a competitive setting since it is important for the incumbent supplier to gain more profit in the future. Then, our study attempts to integrate this topic into the research about the wholesale pricing strategy for an incumbent supplier facing with a competitive supplier and to complement the study about the supplier's pricing strategies in a competitive environment.

## 3. Model Description and the Solutions

In this section, we will introduce the proposed model and its solutions.

### 3.1. Model Description and Formulation

As stated above, for an incumbent supplier who faces with a competitive supplier, if he wants to win the competition with his competitor, he needs to give a wholesale price that is lower than (or at least equal to) his competitor's counterpart, while this lower wholesale price may lead to a loss in his profit from the current deal. In general, to attract the retailer, the competitive supplier wholesale price cannot be known by the incumbent supplier. However, based on the experience or using statistic methods, the incumbent supplier can give a forecast about the distribution of the competitive supplier's wholesale price, and thus the competitive supplier's wholesale price can be seen as a random variable for the incumbent supplier.

Then, we introduce the following profit function *P*(*c*), which considers both the present loss and the future loss from the wholesale price *c* of the incumbent supplier:

(1) $$\begin{array}{c} P\left(c\right)=\left(c-b\right)q-\lambda q{\left(\xi -c\right)}^{+}-2\mu q{\left(c-\xi \right)}^{+}, \end{array}$$

where (*ξ*−*c*)^+^ = max⁡{*ξ* − *c*, 0} and (*c*−*ξ*)^+^ = max⁡{*c* − *ξ*, 0}. In (1), *q* is the order quantity of the retailer, *b* is the producing cost of the incumbent supplier for unit product, *ξ* is a random variable and represents the competitive supplier's wholesale price, *λ* is the deficient penalty coefficient for the incumbent supplier giving a wholesale price which is lower than *ξ*, and *μ* is the excess penalty coefficient for the incumbent supplier giving a wholesale price which is higher than *ξ*. Here, the probability density function and cumulative distribution function of the random variable *ξ* are denoted by *f*(·) and *F*(·), respectively, and it is assumed that *F*(·) is continuously differentiable. Moreover, let *e* and *m* be the minimum and the maximum of the random variable *ξ*, respectively, and thus it satisfies *F*(*e*) = 0 and *F*(*m*) = 1. Then, in the right hand of (1), the first item represents the realized profit of the incumbent supplier in the current deal, the second item represents the incumbent supplier's present loss from the current deal when his wholesale price is lower than his competitor's wholesale price, and the third item represents the incumbent supplier's future loss from losing the retailer when his wholesale price is higher than his competitor's wholesale price. Then, to maximize the profit *P*(*c*), the incumbent supplier had better give a wholesale price that is the same as his competitor's counterpart. For such a case, it follows that *λq*(*ξ*−*c*)^+^ = 0 and *μq*(*c*−*ξ*)^+^ = 0, and both the present loss and the future loss do not exist.

As stated above, it is supposed that if the incumbent supplier's wholesale price *c* is lower than his competitor's counterpart *ξ*, then the retailer will be happy to extend the contract with the incumbent supplier when the contract expires. Otherwise if the incumbent supplier's wholesale price *c* is higher than his competitor's counterpart *ξ*, the retailer will no longer extend the contract with the incumbent supplier and switch to the competitive supplier when the contract expires and there will be a loss in the incumbent supplier's future profit. Thus, in designing his wholesale pricing strategies, the incumbent supplier must consider both the response to his competitor's action and the influence to the retailer.

In practice, if the incumbent supplier pays more attention to the decrease of his present loss in the current deal, he can assign a big value to the deficient penalty coefficient *λ* to reduce his present loss. Otherwise, if the supplier is more concerned with the decrease of his future loss, he can assign a big value to the excess penalty coefficient *μ* to avoid the loss from losing the retailer. Thus, the incumbent supplier can adjust the values *λ* and/or *μ* to balance the present loss and the future loss and maximize his total profit obtained from the current and future cooperations with the retailer.

In the following, we will study the optimal wholesale prices for different objectives about the profit *P*(*c*) for the incumbent supplier.

### 3.2. Optimal Wholesale Price to Maximize the Expected Profit

For the incumbent supplier, since the competitive supplier's wholesale price *ξ* can be seen as a random variable, then the profit *P*(*c*) from his wholesale price *c* is uncertain. Here, facing a random wholesale price of the competitive supplier, a conventional approach is based on assuming the incumbent supplier who makes the wholesale price decision by maximizing his expected profit *E*[*P*(*c*)] (*E* is the expectation operator). For such a case, the incumbent supplier's optimal wholesale price is equal to the optimal solution to the following problem:

$$\begin{array}{cc} \left(\text{P}\right) & \max E\left[P\left(c\right)\right]. \end{array}$$

Then, we have the following result about problem (P).

**Theorem 1 .**

For the incumbent supplier, the optimal wholesale price to maximize his expected profit *E*[*P*(*c*)] is given by

(2) $$\begin{array}{c} c^{*}=F^{-1}\left[\frac{\lambda +1}{\lambda +2\mu }\right]. \end{array}$$

By Theorem 1, if it satisfies *μ* ≤ 0.5, then it follows that (*λ* + 1)/(*λ* + 2*μ*) ≥ 1 and *c* ^*^ will stay at its maximum value. That is to say, if the incumbent supplier assigns a small value to the excess penalty coefficient *μ*, that is, the incumbent supplier pays little/no attention to the future loss from losing the retailer, then he can give a maximum wholesale price to minimize his loss in the current deal. Without loss of generality, it is supposed that *μ* ≥ 0.5 always holds hereinafter. By Theorem 1, the following results are easily obtained.

**Corollary 2 .**

For the incumbent supplier, the optimal wholesale price *c* ^*^ is increasing in the deficient penalty coefficient *λ*.

By this result, if the deficient penalty coefficient *λ* improves, which implies that the incumbent supplier pays more attention to the present loss, then he had better raised his wholesale price to decrease his present loss in the current deal.

**Corollary 3 .**

For the incumbent supplier, the optimal wholesale price *c* ^*^ is decreasing in the excess penalty coefficient *μ*.

By this result, if the excess penalty coefficient *μ* improves, which implies that the incumbent supplier pays more attention to the future loss from losing the retailer, then he had better lowered his wholesale price to encourage the retailer to continue the cooperation between them.

In this subsection, we derive an optimal wholesale price to maximize the expected profit *E*[*P*(*c*)] of the incumbent supplier. It is an acceptable decision if the incumbent supplier is nonsensitive to the profit variations. However, it is known that the significance of the expected performance measure highly depends on its associated variance. Here, if the variance of the profit is large, the chance of deviating from the expected profit will be high and this is not good news for the incumbent supplier who is sensitive to the profit variations. As stated above, since the competition is fierce, the competitive supplier may give a very low wholesale price to attract the retailer. Then, there may be a large fluctuation in the competitive supplier's wholesale price *ξ*, which results in a high variance of the profit *P*(*c*). Evidently, the obtained expected profit maximizing wholesale price *c* ^*^ above fails to reduce/control such a risk and may bring a great loss to the incumbent supplier. Then, to control the risk originating from the fluctuation in the competitive supplier's wholesale price, we will incorporate CVaR measure in finance into the decision framework about the wholesale price for the incumbent supplier in the following subsection.

### 3.3. Optimal Wholesale Price to Maximize the CVaR about Profit

In Section 3.2, we discuss how to choose an optimal wholesale price to maximize the expected profit *E*[*P*(*c*)] of the incumbent supplier. But, this expected profit maximizing wholesale price may bring a great loss to the incumbent supplier since the risk originating from the fluctuation in the competitive supplier's wholesale price is little concerned. Actually, pressures coming from competitions may influence the competitive supplier's decision about the wholesale price, which may lead to a big fluctuation in *ξ*. Evidently, such a fluctuation in *ξ* cannot be neglected by the incumbent supplier in selecting his wholesale price since it may introduce risks and losses. In recent years, managers in practice pay more attention to the risk control problem and a lot of papers have been devoted to the risk analysis in operations and management. In particular, the CVaR measure has been well applied to investigate the pricing and ordering decisions for the supplier/retailer to control the potential risk; see Gotoh and Takano [10] and Chen et al. [11]. Then, to reduce/control the risk originating from the fluctuation in *ξ*, we will integrate the CVaR measure into the decision framework about the wholesale price for the incumbent supplier herein.

For a given confidence level *α*, we first give the definition of VaR about profit *P*(*c*) for the incumbent supplier as follows:

(3) $$\begin{array}{c} \text{Va}{\text{R}}_{\alpha }\left(c\right)=\sup\left\{y\in R\;|\;\mathrm{Pr}\left\{P\left(c\right)\geq y\right\}\geq \alpha \right\}, \end{array}$$

where *Pr*⁡{*P*(*c*) ≥ *y*} denotes the probability of *P*(*c*) that is bigger than the value *y*. Here, the value VaR_*α*_(*c*) represents the maximum profit the incumbent supplier can obtain under the confidence level *α*. Then, taking VaR_*α*_(*c*) as the target profit, the CVaR about profit *P*(*c*) for the incumbent supplier can be defined as

(4) $$\begin{array}{c} \text{CVa}{\text{R}}_{\alpha }\left(c\right)=E\left[P\left(c\right)\;|\;P\left(c\right)\leq \text{Va}{\text{R}}_{\alpha }\left(c\right)\right], \end{array}$$

which represents the expected value of profit *P*(*c*) that is less than the target level VaR_*α*_(*c*). Then, we expect to find an optimal wholesale price for the incumbent supplier to maximize the above CVaR objective, which is equal to the optimal solution to the following problem:

$$\begin{array}{cc} \left({\text{P}}_{\alpha }\right) & \text{maxCVa}{\text{R}}_{\alpha }\left(c\right). \end{array}$$

Here, this CVaR objective pays more attention to the maximization of the profit that is below the target level VaR_*α*_(*c*), while the profit above this target level is not concerned. To reduce the potential risk, the CVaR measure is more appealing than some other risk measures (e.g., the standard deviation measure), since the profit above the target level cannot be regarded as a risk to be hedged but more pleasant gain. Then, we have the following result about the problem (Pα).

**Theorem 4 .**

For the incumbent supplier, the optimal wholesale price to maximize the CVaR objective is given by

(5) cα=1λ+2μ[λF−1((λ+1)+α(2μ−1)λ+2μ)hhhhhhhhh+2μF−1((λ+1)(1−α)λ+2μ)].

It is easily checked that the optimal solution to problem (Pα) is more complicated than that to problem (P). Moreover, if it satisfies *α* = 0, which implies that the incumbent supplier becomes risk neutral and pays no attention to the risk originating from the fluctuation in *ξ*, then *c* ^*α*^ is reduced to *c* ^*^ which is obtained in Section 3.2. Similar to Corollaries 2–3, we have the following result.

**Corollary 5 .**

For the incumbent supplier, the optimal wholesale price *c* ^*α*^ is increasing in the deficient penalty coefficient *λ* and decreasing in the excess penalty coefficient *μ*.

It is pointed out that, the confidence level *α* indicates the risk aversion degree of the incumbent supplier, and the larger the value *α* is, the more risk-averse the incumbent supplier becomes. Then, how does the optimal wholesale price *c* ^*α*^ change with the growth of the confidence level *α* (i.e., the rising of the incumbent supplier's risk aversion degree)? We have the following remark to address this issue.

**Remark 6 .**

For *α* ∈ [0,1), the optimal wholesale price *c* ^*α*^ could be increasing or decreasing in the confidence level *α*. In fact, it follows from Theorem 4 that

(6) cα=1λ+2μ[((λ+1)(1−α)λ+2μ)λF−1((λ+1)+α(2μ−1)λ+2μ)hhhhhhhh+2μF−1((λ+1)(1−α)λ+2μ)].

For simplicity, we denote

(7) $$\begin{array}{c} \frac{\left(\lambda +1\right)+\alpha \left(2\mu -1\right)}{\lambda +2\mu }=\theta ,\;\;\frac{\left(\lambda +1\right)\left(1-\alpha \right)}{\lambda +2\mu }=\kappa . \end{array}$$

It follows that

(8) $$\begin{array}{c} \theta -\kappa =\alpha \geq 0, \end{array}$$

which implies

(9) $$\begin{array}{c} \theta \geq \kappa . \end{array}$$

Then we have

(10) $$\begin{array}{c} c^{\alpha }=\frac{1}{\lambda +2\mu }\left[\lambda F^{-1}\left(\theta \right)+2\mu F^{-1}\left(\kappa \right)\right], \end{array}$$

(11) ∂cα∂α=1(λ+2μ)2[2λμ−λf[F−1(θ)]−2λμ+2μf[F−1(κ)]]=(2λμ−λ)f[F−1(κ)]−(2λμ+2μ)f[F−1(θ)](λ+2μ)2f[F−1(θ)]f[F−1(κ)].

Equation (11) shows that the sign of ∂*c* ^*α*^/∂*α* is the same as the sign of (2*λμ* − *λ*)*f*[*F* ^−1^(*κ*)]−(2*λμ* + 2*μ*)*f*[*F* ^−1^(*θ*)], which can be positive or negative. This shows that the optimal wholesale price *c* ^*α*^ could be increasing or decreasing in the confidence level *α*. Specially, if *f*(·) is increasing, it concludes from *θ* ≥ *κ* that *f*[*F* ^−1^(*θ*)] ≥ *f*[*F* ^−1^(*κ*)]; then it follows that (2*λμ* − *λ*)*f*[*F* ^−1^(*κ*)]−(2*λμ* + 2*μ*)*f*[*F* ^−1^(*θ*)] ≤ 0, which implies ∂*c* ^*α*^/∂*α* ≤ 0 by (11), and then the optimal wholesale price *c* ^*α*^ is then decreasing in the confidence level *α*.

By this remark, if the incumbent supplier becomes more risk-averse, the optimal wholesale price *c* ^*α*^ may be increasing or decreasing, and thus the incumbent supplier may give a higher or lower wholesale price than the optimal wholesale price *c* ^*^. It can be explained as follows: if the incumbent supplier becomes more risk-averse, he is more sensitive to the loss variations, since his present loss from a lower wholesale price and the future loss from a higher wholesale price are not equal, and the term that dominates determines the direction of changes to the optimal wholesale price *c* ^*α*^.

As stated above, if the confidence level *α* becomes larger, the incumbent supplier will face a lower risk. Then how does the expected profit *E*[*P*(*c* ^*α*^)] under the optimal wholesale price *c* ^*α*^ for the incumbent supplier change with the growth of *α*? We have the following result.

**Theorem 7 .**

For *α* ∈ [0,1), the expected profit *E*[*P*(*c* ^*α*^)] under the optimal wholesale price *c* ^*α*^ for the incumbent supplier is decreasing in the confidence level *α*.

This result shows that if the incumbent supplier hopes a lower risk, he will expect a lower profit. This verifies the fact that low risk implies low return, while with high risk comes high return.

In this subsection, to reduce/control the risk originating from the fluctuation in the competitive supplier's wholesale price, we introduce the CVaR measure into the decision framework about the wholesale price for the incumbent supplier. The optimal wholesale price *c* ^*α*^ to the CVaR objective can maximize the expectation of the profit that is below the given target level, which can help to reduce the downside risk for the incumbent supplier. Moreover, since the confidence level *α* characterizes the risk level, the incumbent supplier can control the risk level he faces by adjusting the value of *α*. If the incumbent supplier becomes more risk-averse, he can assign a big value to the confidence level *α*; otherwise he can assign a small value to the confidence level *α*.

## 4. Numerical Example

In this section, we will give a numerical example to illustrate the obtained results.

**Example 8 .**

**Figure 1:**
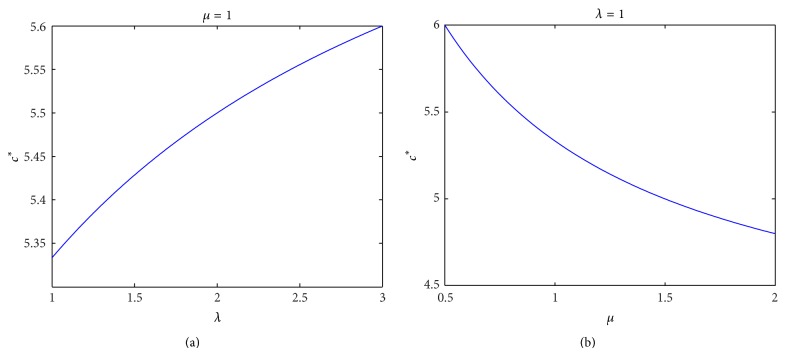
The optimal wholesale price *c* ^*^ with different parameters *λ* and *μ*.

**Figure 2:**
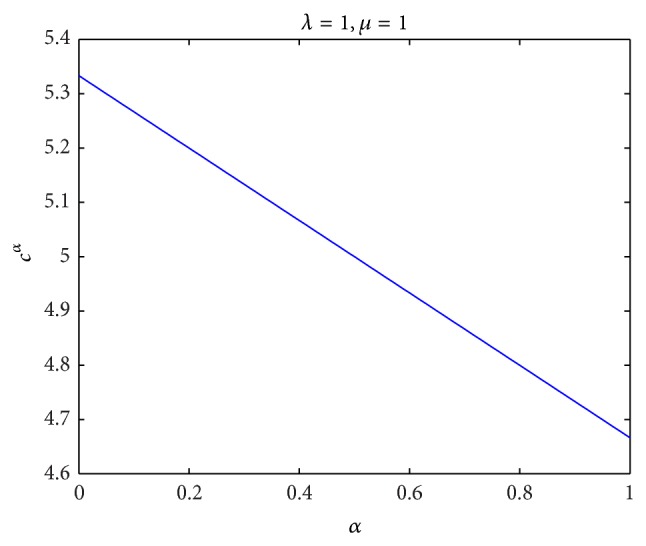
The optimal wholesale price *c* ^*α*^ with different confidence level *α*.

**Figure 3:**
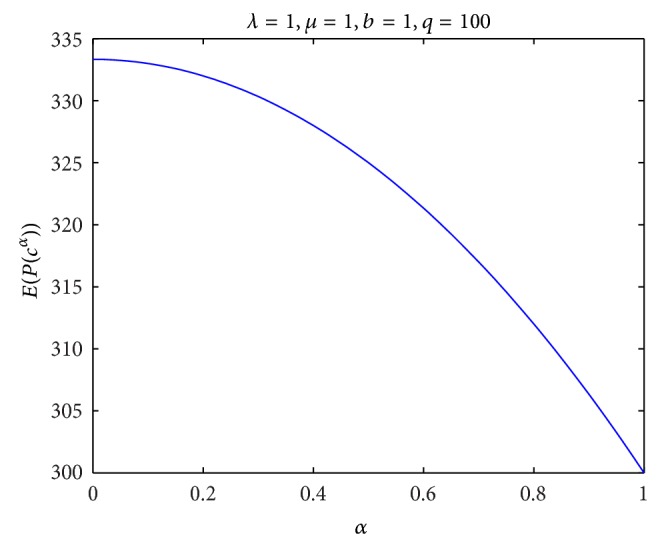
Expected profit *E*[*P*(*c* ^*α*^)] with different confidence level *α*.

For a two-stage supply chain, suppose that the competitive supplier's wholesale price *ξ* is subject to uniform distribution *U*(4,6). For different parameters, let us compute the optimal wholesale prices *c* ^*^ and *c* ^*α*^ for the incumbent supplier and give some sensitivity analysis. First, for fixed *μ* and *λ*, respectively, we compute the optimal wholesale price *c* ^*^ with different parameters *λ* and *μ*, respectively, and the results are given in Figure 1. Then, for *λ* = 1 and *μ* = 1, we compute the optimal wholesale price *c* ^*α*^ with different confidence level *α*, and the result is given in Figure 2. Finally, for *λ* = 1, *μ* = 1, *b* = 1, and *q* = 100, we compute the expected profit *E*[*P*(*c* ^*α*^)] with different confidence level *α*, and the result is given in Figure 3. By Figures 1, 2, and 3, the numerical results confirm the obtained results in Section 3.

## 5. Conclusions

The growing emphasis on globalization and rapid technological advancements have intensified business competition; there are often multiple suppliers that compete for the same customer. Moreover, it is not surprising to see that more and more suppliers resort to dependence on customer services and product quality to win this competition. However, for the competition of the same/similar product, price advantage evidently plays a prominent role in capturing more customers for a supplier. Thus, designing adaptive pricing strategies for a supplier to compete with other suppliers comes into focus. With regret, the existing pricing strategies for such cases always pay more attention to the profit maximization or cost minimization of the supplier, while continuing the cooperation with the retailers, which guarantee the future profit of the supplier is often neglected.

This paper investigates the optimal wholesale pricing strategy of an incumbent supplier who confronts the competition from a competitive supplier. We introduce a profit function, which considers not only the present loss in the current deal but also the future loss from losing the retailer for the incumbent supplier. Then, we achieve the optimal wholesale prices that optimize different objectives about this profit function for the incumbent supplier. Then, an incumbent supplier can choose a proper objective by his own preference. Our study thus provides a practical wholesale pricing strategy for an incumbent supplier in a competitive setting.

## Appendix

### Proof of the Results

Proof of Theorem 1. For a given wholesale price *c* of the incumbent supplier and a realized value *z* of *ξ*, by (1), we have

(A.1) $$\begin{array}{c} P\left(c\right)=\left(c-b\right)q-\lambda q{\left(z-c\right)}^{+}-2\mu q{\left(c-z\right)}^{+}; \end{array}$$

it follows from (*z*−*c*)^+^ = (*z* − *c*) + (*c*−*z*)^+^ that

(A.2) $$\begin{array}{c} P\left(c\right)=\left(\lambda +1\right)qc-\lambda qz-bq-\left(\lambda +2\mu \right)q{\left(c-z\right)}^{+}. \end{array}$$

Then the expectation of *P*(*c*) is given by

(A.3) E[P(c)]=(λ+1)qc−λqE(ξ)−bq −(λ+2μ)q∫ec(c−t)dF(t);

it follows from *F*(*e*) = 0 that

(A.4) $$\begin{array}{c} \frac{\partial E\left[P\left(c\right)\right]}{\partial c}=\left(\lambda +1\right)q-\left(\lambda +2\mu \right)qF\left(c\right). \end{array}$$

Further, it follows that

(A.5) $$\begin{array}{c} \frac{\partial^{2}E\left[P\left(c\right)\right]}{\partial c^{2}}=-\left(\lambda +2\mu \right)qf\left(c\right)<0, \end{array}$$

which implies that *E*[*P*(*c*)] is concave in *c*. Then it follows from (A.4) and ∂*E*[*P*(*c*)]/∂*c* = 0 that *E*[*P*(*c*)] attains the maximum in

(A.6) $$\begin{array}{c} c^{*}=F^{-1}\left[\frac{\lambda +1}{\lambda +2\mu }\right]. \end{array}$$

This completes the proof.

Proof of Theorem 4. By (A.2), we have

(A.7) $$\begin{array}{c} P\left(c\right)=\left(\lambda +1\right)qc-\lambda qz-bq-\left(\lambda +2\mu \right)q{\left(c-z\right)}^{+}. \end{array}$$

Now, we define an auxiliary function

(A.8) h(c,v)=v−11−αE[v−P(c)]+=v−11−α∫em[v−(λ+1)qc+λqt+bqhhhhhhhhhhhhhh+(λ+2μ)q(c−t)+]+dF(t)=v−11−α∫ec[v+bq+(2μ−1)qc−2μqt]+dF(t) −11−α∫cm[v+bq−(λ+1)qc+λqt]+dF(t).

By the result in Rockafellar and Uryasev [12] (Section 3, Corollary 11), *h*(*c*, *v*) is jointly concave in (*c*, *v*) since *P*(*c*) is concave in *c*. Further, by the result in Rockafellar and Uryasev [13], the optimal solution *c* ^*α*^ to problem (Pα) is equal to the optimal solution to the following problem:

(A.9) $$\begin{array}{c} \max\left[\underset{v\in R}{\max}\;h\left(c,v\right)\right]. \end{array}$$

Then, for any fixed *c*, we first solve the optimal solution *v* ^*^ to the problem max⁡_*v*∈*R*_ *h*(*c*, *v*). We distinguish the following cases. (i) Consider *v* ≤ *q*(1 − 2*μ*)(*c* − *b*). In this case, by (A.8), we have

(A.10) h(c,v)=v−11−α ×∫((λ+1)qc−bq−v)/λqm[v+bq−(λ+1)qc+λqt]dF(t)

and thus

(A.11) $$\begin{array}{c} \frac{\partial h\left(c,v\right)}{\partial v}=1-\frac{1}{1-\alpha }\left[1-F\left(\frac{\left(\lambda +1\right)qc-bq-v}{\lambda q}\right)\right]. \end{array}$$

Obviously, when *v* is sufficiently small (*v* < (*λ* + 1)*qc* − *bq* − *λqm*), it follows from (A.11) that ∂*h*(*c*, *v*)/∂*c* = 1 > 0 holds. Then, if it satisfies (∂*h*(*c*, *v*)/∂*c*)|_*v*=*q*(1−2*μ*)(*c*−*b*)_ = 1 − (1/(1 − *α*))[1 − *F*(((*λ* + 2*μ*)*c* − 2*μb*)/*λ*)] ≤ 0, which implies *c* ≤ (2*μb* + *λF* ^−1^(*α*))/(*λ* + 2*μ*), it follows from (A.11) that the optimal solution *v* ^*^ to problem max⁡_*v*∈*R*_ *h*(*c*, *v*) solves

(A.12) $$\begin{array}{c} 1-\frac{1}{1-\alpha }\left[1-F\left(\frac{\left(\lambda +1\right)qc-bq-v^{*}}{\lambda q}\right)\right]=0, \end{array}$$

which implies

(A.13) $$\begin{array}{c} v^{*}=\left(\lambda +1\right)qc-bq-\lambda qF^{-1}\left(\alpha \right). \end{array}$$

(ii) Consider *q*(1 − 2*μ*)(*c* − *b*) < *v* ≤ *q*(*c* − *b*). In this case, by (A.8), we have

(A.14) h(c,v)=v−11−α ×∫e(v+bq+(2μ−1)qc)/2μq[v+bq+(2μ−1)qc−2μqt]dF(t) −11−α ×∫((λ+1)qc−bq−v)/λqm[v+bq−(λ+1)qc+λqt]dF(t)

and thus

(A.15) ∂h(c,v)∂v=1−11−α[1+F(v+bq+(2μ−1)qc2μq)hhhhhhhhhh−F((λ+1)qc−bq−vλq)].

Obviously, it follows from (A.15) that (∂*h*(*c*, *v*)/∂*v*)|_*v*=*q*(*c*−*b*)_ = 1 − 1/(1 − *α*) < 0 holds. Then, if it satisfies (∂*h*(*c*, *v*)/∂*c*)|_*v*=*q*(1−2*μ*)(*c*−*b*)_ = 1 − 1/(1 − *α*)[1 − *F*(((*λ* + 2*μ*)*c* − 2*μb*)/*λ*)] ≥ 0, which implies *c* ≥ (2*μb* + *λF* ^−1^  (*α*))/(*λ* + 2*μ*), it follows from (A.15) that the optimal solution *v* ^*^ to max⁡_*v*∈*R*_ *h*(*c*, *v*) solves

(A.16) 1−11−α[1+F(v∗+bq+(2μ−1)qc2μq)hhhhhhhh−F((λ+1)qc−bq−v∗λq)]=0.

(iii) Consider *v* > *q*(*c* − *b*). In this case, by (A.8), we have

(A.17) h(c,v)=v−11−α∫ec[v+bq+(2μ−1)qc−2μqt]dF(t) −11−α∫cm[v+bq−(λ+1)qc+λqt]dF(t)

and thus

(A.18) $$\begin{array}{c} \frac{\partial h\left(c,v\right)}{\partial v}=1-\frac{1}{1-\alpha }<0. \end{array}$$

Based on the analysis above, it is clear that, for any fixed *c*, *h*(*c*, *v*) attains the maximum when *v* ≤ *q*(*c* − *b*). Further, for any fixed *c*, the optimal solution *v* ^*^ to problem max⁡_*v*∈*R*_ *h*(*c*, *v*) is given by

(A.19) $$\begin{array}{c} v^{*}=\{\begin{array}{cc} \left(\lambda +1\right)qc-bq-\lambda qF^{-1}\left(\alpha \right) & c\leq \frac{2\mu b+\lambda F^{-1}\left(\alpha \right)}{\lambda +2\mu },\\ v^{1} & c\geq \frac{2\mu b+\lambda F^{-1}\left(\alpha \right)}{\lambda +2\mu }, \end{array} \end{array}$$

where *v* ^1^ solves (A.16). Next, to solve the problem max⁡[max⁡_*v*∈*R*_ *h*(*c*, *v*)] = max⁡*h*(*c*, *v* ^*^), we distinguish between the following cases. (a) Consider *c* ≤ (*μb* + *λF* ^−1^(*α*))/(*λ* + *μ*). In this case, the optimal solution *v* ^*^ to problem max⁡_*v*∈*R*_ *h*(*c*, *v*) is given by

(A.20) $$\begin{array}{c} v^{*}=\left(\lambda +1\right)qc-bq-\lambda qF^{-1}\left(\alpha \right). \end{array}$$

Then by (A.8), we have

(A.21) h(c,v∗) =v∗−11−α  ×∫((λ+1)qc−bq−v∗)/λqm[v∗+bq−(λ+1)qc+λqt]dF(t) =(λ+1)qc−bq−λqF−1(α)−11−α  ×∫F−1(α)mλq(t−F−1(α))dF(t)

and thus

(A.22) $$\begin{array}{c} \frac{\partial h\left(c,v^{*}\right)}{\partial c}=\left(\lambda +1\right)q>0. \end{array}$$

(b) Consider *c* ≥ (*μb* + *λF* ^−1^(*α*))/(*λ* + 2*μ*). In this case, the optimal solution *v* ^*^ to problem max⁡_*v*∈*R*_ *h*(*c*, *v*) is given by *v* ^*^ = *v* ^1^, where *v* ^1^ solves (A.16). By (A.16), it follows that

(A.23) $$\begin{array}{c} F\left[\frac{\left(\lambda +1\right)qc-bq-v^{1}}{\lambda q}\right]-F\left[\frac{v^{1}+bq+\left(2\mu -1\right)qc}{2\mu q}\right]=\alpha . \end{array}$$

Then by (A.8), we have

(A.24) h(c,v∗)=h(c,v1)=v1−11−α ×∫e(v1+bq+(2μ−1)qc)/2μq[v1+bq+(2μ−1)qc−2μqt]dF(t) −11−α ×∫((λ+1)qc−bq−v1)/λqm[v1+bq−(λ+1)qc+λqt]dF(t)

and thus

(A.25) ∂h(c,v1)∂c=−(2μ−1)q1−αF[v1+bq+(2μ−1)qc2μq] +(λ+1)q1−α[1−F((λ+1)qc−bq−v1λq)].

It is obvious that the optimal solution *c* ^*α*^ to problem max⁡*h*(*c*, *v* ^1^) is given by ∂*h*(*c*, *v* ^1^)/∂*c* = 0; then it follows from (A.25) that *c* ^*α*^ satisfies

(A.26) −(2μ−1)F[v1+bq+(2μ−1)qcα2μq]  +(λ+1)[1−F((λ+1)cα−bq−v1λq)]=0.

Then it follows from (A.23) and (A.26) that

(A.27) $$\begin{array}{c} F\left[\frac{v^{1}+bq+\left(2\mu -1\right)qc^{\alpha }}{2\mu q}\right]=\frac{\left(\lambda +1\right)\left(1-\alpha \right)}{\lambda +2\mu }, \end{array}$$

which implies

(A.28) $$\begin{array}{c} v^{1}+bq+\left(2\mu -1\right)qc^{\alpha }=2\mu qF^{-1}\left[\frac{\left(\lambda +1\right)\left(1-\alpha \right)}{\lambda +2\mu }\right]. \end{array}$$

Moreover, it follows from (A.23) and (A.27) that

(A.29) $$\begin{array}{c} F\left[\frac{\left(\lambda +1\right)qc^{\alpha }-bq-v^{1}}{\lambda q}\right]=\frac{\left(\lambda +1\right)+\alpha \left(2\mu -1\right)}{\lambda +2\mu }, \end{array}$$

which implies

(A.30) $$\begin{array}{c} \left(\lambda +1\right)qc^{\alpha }-bq-v^{1}=\lambda qF^{-1}\left[\frac{\left(\lambda +1\right)+\alpha \left(2\mu -1\right)}{\lambda +2\mu }\right]. \end{array}$$

Then, it follows form (A.28) and (A.30) that

(A.31) cα=1λ+2μ[λF−1((λ+1)+α(2μ−1)λ+2μ)hhhhhhhhh+2μF−1((λ+1)(1−α)λ+2μ)].

This completes the proof.

Proof of Corollary 5. By Theorem 4, the optimal wholesale price *c* ^*α*^ is given by

(A.32) cα=1λ+2μ[λF−1((λ+1)+α(2μ−1)λ+2μ)hhhhhhhhh+2μF−1((λ+1)(1−α)λ+2μ)]=1λ+2μ[λF−1(θ)+2μF−1(κ)].

Then it follows that

(A.33) ∂cα∂λ=(∂[λF−1(θ)+2μF−1(κ)]/∂λ)(λ+2μ)(λ+2μ)2 −λF−1(θ)+2μF−1(κ)(λ+2μ)2=(((1−α)(2μ−1)[λ(f(F−1  (θ)))−1hhhhhhhhhhhhhihhh+2μ(f(F−1  (κ)))−1])hhh×(λ+2μ)−1+(λ+2μ)F−1(θ)((1−α)(2μ−1)[λ(f(F−1  (θ)))−1)((λ+2μ)2)−1 −λF−1(θ)+2μF−1(κ)(λ+2μ)2=((1−α)(2μ−1)[λ(f(F−1(θ)))−1hhhhhhhhhhhhihhh+2μ(f(F−1(κ)))−1])hhh×((λ+2μ)3)−1 +2μ(λ+2μ)2[F−1(θ)−F−1(κ)].

It follows from *θ* ≥ *κ* that *F* ^−1^(*θ*) − *F* ^−1^(*κ*) ≥ 0 holds, which implies ∂*c* ^*α*^/∂*λ* ≥ 0 by (A.33); this proves that *c* ^*α*^ is increasing in the deficient penalty coefficient *λ*. Similarly, we can prove that *c* ^*α*^ is decreasing in the excess penalty coefficient *μ*. This completes the proof.

Proof of Theorem 7. By (A.4), we have

(A.34) $$\begin{array}{c} \frac{\partial E\left[P\left(c\right)\right]}{\partial c}=\left(\lambda +1\right)q-\left(\lambda +2\mu \right)qF\left(c\right). \end{array}$$

Then it follows that

(A.35) $$\begin{array}{c} \frac{\partial E\left[P\left(c^{\alpha }\right)\right]}{\partial \alpha }=\left[\left(\lambda +1\right)q-\left(\lambda +2\mu \right)qF\left(c^{\alpha }\right)\right]\frac{\partial c^{\alpha }}{\partial \alpha }. \end{array}$$

By Remark 6, if *c* ^*α*^ is increasing in the confidence level *α*, we have ∂*c* ^*α*^/∂*α* ≥ 0 and *c* ^*α*^ ≥ *c* ^*^. Then it follows from *c* ^*^ = *F* ^−1^[(*λ* + 1)/(*λ* + 2*μ*)] that

(A.36) (λ+1)q−(λ+2μ)qF(cα) ≤(λ+1)q−(λ+2μ)qF(c∗)=0.

It follows from (A.35), (A.36), and ∂*c* ^*α*^/∂*α* ≥ 0 that

(A.37) $$\begin{array}{c} \frac{\partial E\left[P\left(c^{\alpha }\right)\right]}{\partial \alpha }\leq 0, \end{array}$$

which proves that *E*[*P*(*c* ^*α*^)] is decreasing in the confidence level *α*. Otherwise, if *c* ^*α*^ is decreasing in the confidence level *α*, we have ∂*c* ^*α*^/∂*α* ≤ 0 and *c* ^*α*^ ≤ *c* ^*^. Then it follows from *c* ^*^ = *F* ^−1^[(*λ* + 1)/(*λ* + 2*μ*)] that

(A.38) (λ+1)q−(λ+2μ)qF(cα) ≥(λ+1)q−(λ+2μ)qF(c∗)=0.

It follows from (A.35), (A.38), and ∂*c* ^*α*^/∂*α* ≤ 0 that

(A.39) $$\begin{array}{c} \frac{\partial E\left[P\left(c^{\alpha }\right)\right]}{\partial \alpha }\leq 0, \end{array}$$

which also proves that *E*[*P*(*c* ^*α*^)] is decreasing in the confidence level *α*. This completes the proof.

## References

[1] Bernstein F., Federgruen A. (2003). Pricing and replenishment strategies in a distribution system with competing retailers. *Operations Research*.

[2] Choudhary V., Ghose A., Mukhopadhyay T., Rajan U. (2005). Personalized pricing and quality differentiation. *Management Science*.

[3] Ma L., Zhang R., Guo S., Liu B. (2012). Pricing decisions and strategies selection of dominant manufacturer in dual-channel supply chain. *Economic Modelling*.

[4] Dai Y., Chao X., Fang S.-C., Nuttle H. L. W. (2005). Pricing in revenue management for multiple firms competing for customers. *International Journal of Production Economics*.

[5] Sohn S. Y., Moon T. H., Seok K. J. (2009). Optimal pricing for mobile manufacturers in competitive market using genetic algorithm. *Expert Systems with Applications*.

[6] Xiao T., Qi X. (2010). Strategic wholesale pricing in a supply chain with a potential entrant. *European Journal of Operational Research*.

[7] Li J., Wang S., Cheng T. C. E. (2010). Competition and cooperation in a single-retailer two-supplier supply chain with supply disruption. *International Journal of Production Economics*.

[8] Xia Y. (2011). Competitive strategies and market segmentation for suppliers with substitutable products. *European Journal of Operational Research*.

[9] Wang J.-C., Wang A.-M., Wang Y.-Y. (2013). Markup pricing strategies between a dominant retailer and competitive manufacturers. *Computers & Industrial Engineering*.

[10] Gotoh J.-Y., Takano Y. (2007). Newsvendor solutions via conditional value-at-risk minimization. *European Journal of Operational Research*.

[11] Chen Y., Xu M., Zhang Z. G. (2009). A risk-averse newsvendor model under the CVaR criterion. *Operations Research*.

[12] Rockafellar R. T., Uryasev S. (2002). Conditional value-at-risk for general loss distributions. *Journal of Banking and Finance*.

[13] Rockafellar R. T., Uryasev S. (2000). Optimization of conditional value-at-risk. *The Journal of Risk*.

